# *TP53* co-mutations in advanced lung adenocarcinoma: comparative bioinformatic analyses suggest ambivalent character on overall survival alongside *KRAS*, *STK11* and *KEAP1* mutations

**DOI:** 10.3389/fonc.2024.1357583

**Published:** 2024-04-22

**Authors:** Armin Frille, Myriam Boeschen, Hubert Wirtz, Mathias Stiller, Hendrik Bläker, Maximilian von Laffert

**Affiliations:** ^1^ Department of Respiratory Medicine, Leipzig University, Leipzig, Germany; ^2^ Institute of Pathology, Leipzig University, Leipzig, Germany

**Keywords:** NSCLC, lung adenocarcinoma, *KRAS*, *STK11*, *KEAP1*, *TP53*, co-mutations, survival

## Abstract

**Background:**

Recently, we could show that the co-mutations of *KRAS* + *KEAP1*, *STK11* + *KEAP1* and *KRAS* + *STK11* + *KEAP1* lead to a significantly shorter median overall survival (mOS) in patients with lung cancer across treatments by analyzing multiple dataset. *TP53*, a tumor suppressor gene, plays a crucial role in regulating cell cycle progression. Its mutations occur in approximately 40-50% of non-small lung cancer (NSCLC). Co-occurrence of all four mentioned mutations has been a matter of debate for years. The aim of this study was to assess the distribution of these four mutations and the influence of the different co-mutational patterns on survival.

**Methods:**

We present a comparative bioinformatic analysis and refer to data of 4,109 patients with lung adenocarcinoma (LUAD).

**Results:**

Most of the mutations within the LUAD belong to *TP53*-only (29.0%), quadruple-negative (25.9%) and *KRAS*-only (13.4%). Whereas TP53-mutations seem to have protective effects in the context of further *KEAP1*- and *KRAS* + *KEAP1*-alterations (improved mOS), their role seems contrary if acquired in an already existing combination of mutations as *KRAS* + *STK11*, *KRAS* + *STK11* + *KEAP1* and *STK11* + *KEAP1*. *TP53* co-mutations had a negative influence on *KRAS*-only mutated LUAD (mOS reduced significantly by more than 30%).

**Discussion:**

These data underline the need for complex mutational testing to estimate prognosis more accurately in patients with advanced LUAD.

## 1 Introduction

Lung Cancer is the leading cause of cancer death worldwide, with non-small lung cancer (NSCLC) representing the largest group. Lung adenocarcinoma (LUAD) belongs to the most common and best studied histological subgroups (1). Besides the common treatment strategies consisting of surgery, radiation and chemotherapy, the development and approval of targeted therapies and immune checkpoint inhibitors (ICI) explicitly improved therapy options and patients’ outcome within the past decade. However, treatment responses still vary in a wide range even for the personalized treatment options available (2). Therefore, amongst others, one major need is to acknowledge the significance of genetic co-alterations and their influence on therapy responses.

*KRAS* (Kirsten rat sarcoma viral oncogene homolog) plays a key role in the development and progression of various cancers, including NSCLC. Mutations occur in about 25% of cases, leading to the constitutive activation of *KRAS* signaling pathways, promoting uncontrolled cell growth and survival (3). *KRAS*-altered NSCLC frequently show co-mutations within the genes Kelch-like ECH-associated protein 1 (*KEAP1*) and serine/threonine kinase 11 (*STK11*), also known as liver kinase B1 (*LKB1*) (4). Recently, we could show, by analyzing multiple datasets, that the co-mutations of *KRAS* + *KEAP1*, *STK11* + *KEAP1* and *KRAS* + *STK11* + *KEAP1* lead to a significantly shorter median overall survival (mOS) across treatments. In contrast, patients with tumors harboring only *KRAS* mutations or being negative for all above-mentioned alterations show a significantly improved mOS in a multivariate analysis. Furthermore, triple co-mutated primary tumors showed a significantly increased frequency of distant metastases to bone and adrenal glands (5). Thus, analysis of the complex mutational network seems inevitable in the clinical routine setting.

*TP53* (tumor protein p53), a tumor suppressor gene, plays a crucial role in regulating cell cycle progression, DNA repair, as well as apoptosis and is one of the most common alterations among all cancers, and among LUAD in particular (6). Its mutations occur in approximately 40-50% of NSCLC cases, leading mainly to a loss of function, allowing cells to evade normal regulatory mechanisms and promoting tumorigenesis (7).

Co-occurrence of all four mentioned mutations has been a matter of debate for years: Aredo et al. (8) described concurrent pathogenic mutations of *KRAS* with *TP53* (39%), *STK11* (12%) and *KEAP1* (8%), discussing distinct molecular subtypes (study with a total of 186 patients). Furthermore, they could show that combined **KRAS* G12C* and *TP53* mutations predict benefit from immunotherapy.

Frost et al. focused on 119 patients with lung adenocarcinoma receiving pembrolizumab monotherapy as first-line palliative treatment. Here, rates for *KRAS*, *TP53* and combined mutations were 52.1%, 47.1% and 21.9%, respectively. Whereas, *TP53* mutations alone had no impact on response and survival, a subgroup (12 patients) with *KRAS G12C* + *TP53* co-mutations defined long-term responders to immunotherapy (9).

Recently, Proulx-Rocray et al. described 100 patients with known *KRAS* status. They postulated that *KRAS* mutation in NSCLC might be associated with a favorable response to ICI therapy in the absence of a concurrent mutation in the *STK11* and/or *KEAP1* tumor suppressor genes (10). A survival advantage associated with *TP53* mutation in NSCLC treated with ICIs has been reported in current literature (9, 11–13).

However, the above-mentioned studies only encompass a low number of patients. Furthermore, besides ICI, “classical” chemotherapy still presents the main cornerstone of therapy. Therefore, we here present a comprehensive bioinformatic analysis encompassing two datasets retrieved from cBioPortal and tested the influence of *TP53* co-mutations depending on the *KRAS*, *STK11* and *KEAP1* status.

The aim of this study was to assess the distribution of the four mutations **KRAS*, *STK11*, *KEAP1**, and *TP53* as single mutations as well as co-mutations in patients with advanced LUAD in a large dataset. Furthermore, we want to study the influence of the different co-mutational combinations on survival.

## 2 Materials and methods

For this study, the following two datasets from the MSK institute were retrieved from cBioPortal (14, 15): the “MSK-IMPACT Clinical Sequencing Cohort (MSKCC, Nat Med 2017)” (16) and the “MSK MetTropism (MSK, Cell 2021)” dataset (17). To create one dataset across treatments the two datasets were merged into one “MSK across treatments” dataset (N = 4,855 NSCLC patients, 4,109 LUAD patients, 542 lung squamous cell carcinoma (LSCC) patients). Therapy details were not annotated. Due to the more recent data, data from the “MSK MetTropism (MSK, Cell 2021)” were preferred over the “MSK-IMPACT Clinical Sequencing Cohort (MSKCC, Nat Med 2017)”. All data comprised patients with advanced tumor stages (mainly stage IV). Analyses were performed on LUAD data.

**Figure 1:**
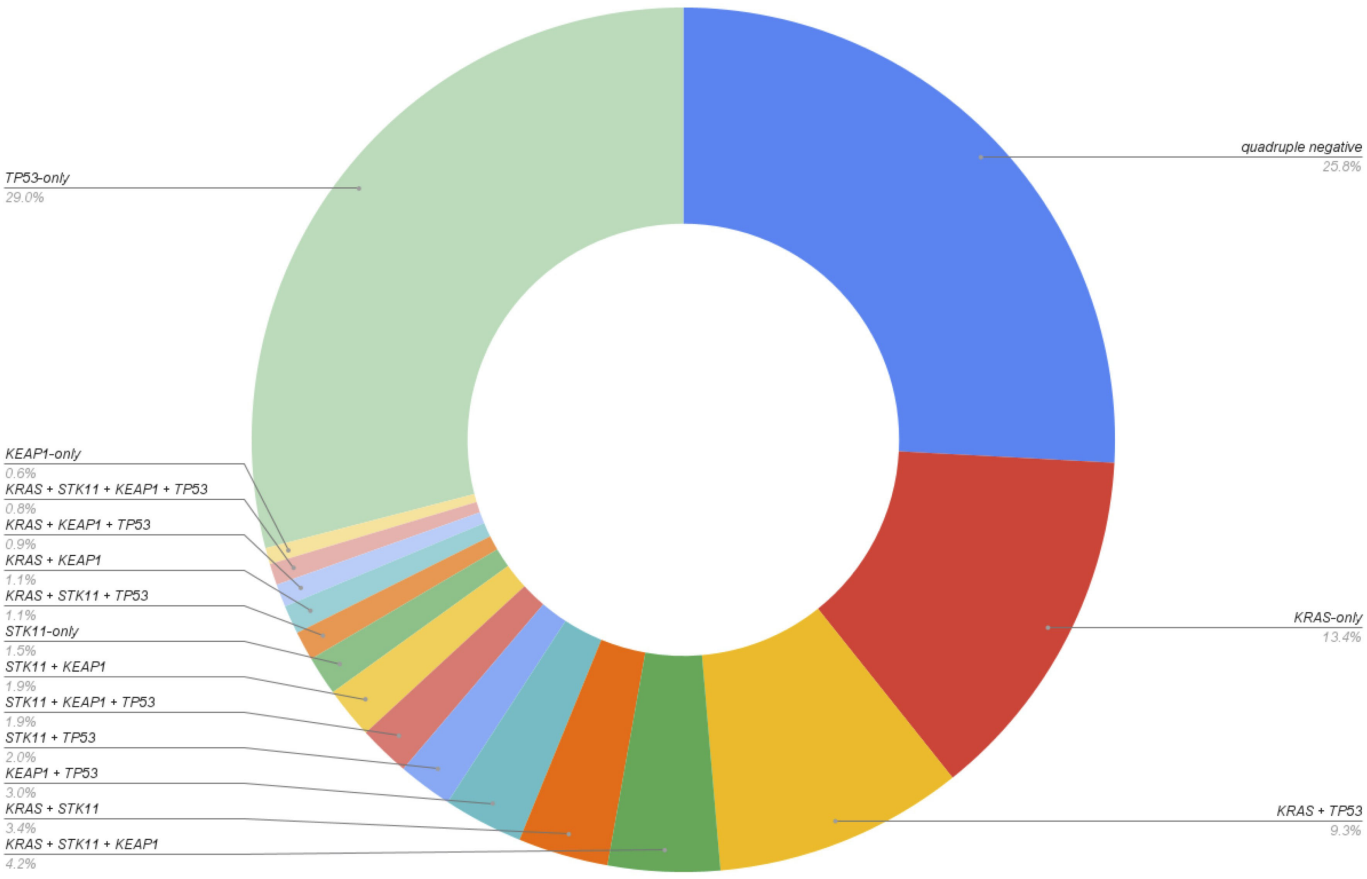
Distribution of mutation groups in the LUAD dataset (N=4,109).

**Figure 2:**
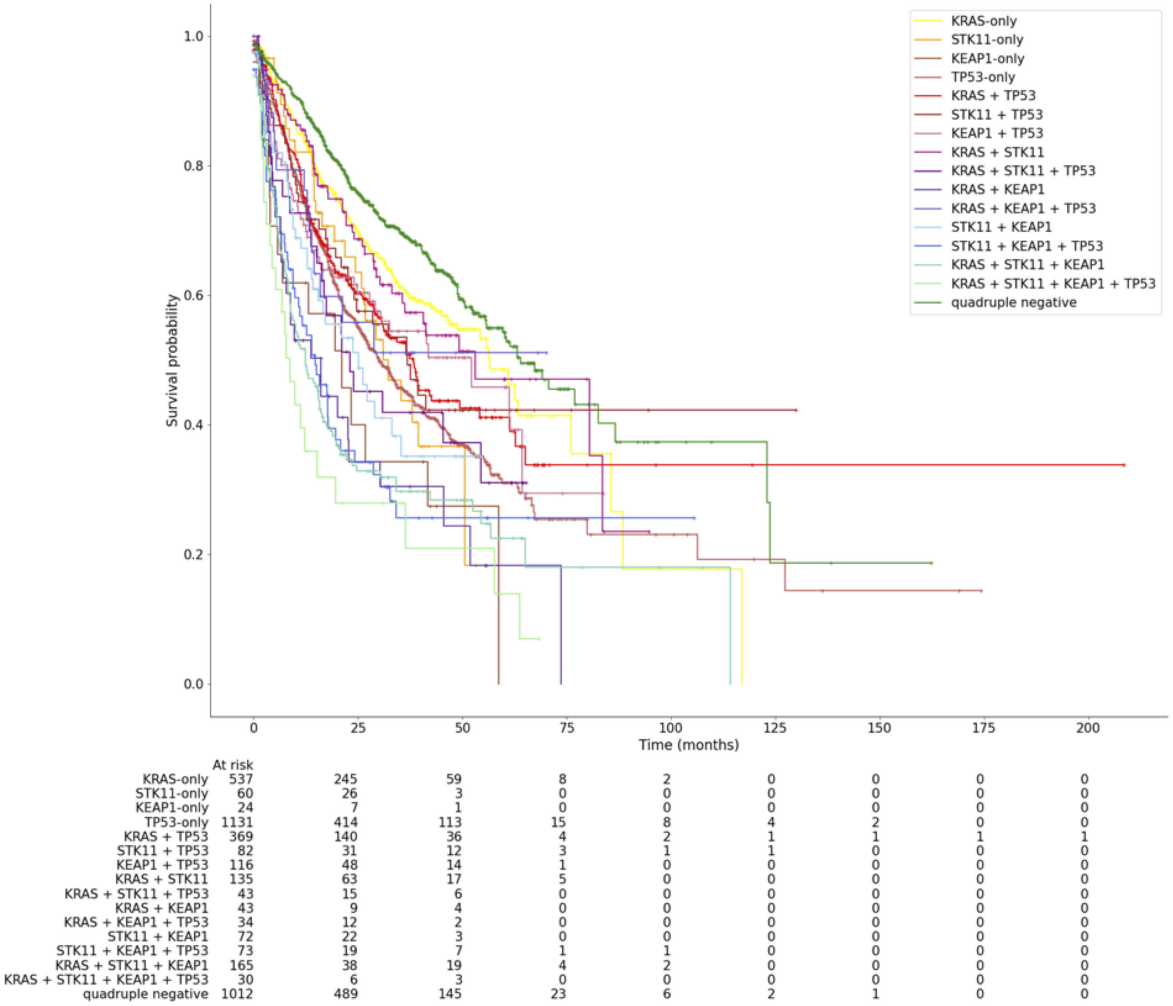
Kaplan Meier curve showing overall survival of LUAD patients among all mutation groups.

In total, 16 combinatory groups of patients were established based on the four genes *KRAS*, *STK11*, *KEAP1*, and *TP53* (**Table 1**, **Figures 1**, **2**): quadruple negative, *KRAS*-only, *STK11*-only, *KEAP1*-only, *TP53*-only, *KRAS* + *TP53*, *STK11* + *TP53*, *KEAP1* + *TP53*, *KRAS* + *STK11*, *KRAS* + *STK11* + *TP53*, *KRAS* + *KEAP1*, *KRAS* + *KEAP1* + *TP53*, *STK11* + *KEAP1*, *STK11* + *KEAP1* + *TP53*, *KRAS* + *STK11* + *KEAP1*, *KRAS* + *STK11* + *KEAP1* + *TP53*.

**Table 1:** Distribution of co-mutations in the genes of **KRAS*, *STK11*, *KEAP1**, and *TP53*, and overall survival in patients with LUAD.

(Co-) mutations	LUAD				
	N	%	mOS (months)	lower 95% CI (months)	upper 95% CI (months)
**Total**	**4,109**	**100**			
*TP53*-only	1,193	29.0	30.0	26.88	33.96
Quadruple-negative	1,062	25.9	64.0	59.64	82.56
*KRAS*-only	552	13.4	56.5	46.8	76.08
*KRAS* + *TP53*	384	9.4	38.3	30.72	49.44
*KEAP1* + *TP53*	123	3.0	52.2	27.84	NR
*KRAS* + *STK11* + *KEAP1*	172	4.2	12.4	8.88	16.08
*KRAS* + *STK11*	139	3.4	53.0	35.64	83.64
*STK11* + *TP53*	84	2.0	36.8	23.4	NR
*STK11* + *KEAP1* + *TP53*	79	1.9	14.8	9.36	19.56
*STK11* + *KEAP1*	79	1.9	25.1	13.8	35.28
*STK11*-only	60	1.5	32.3	24.36	50.52
*KRAS* + *STK11* + *TP53*	45	1.1	23.0	15.12	54.36
*KRAS* + *KEAP1*	44	1.1	16.1	6.6	22.8
*KRAS* + *KEAP1* + *TP53*	35	0.9	NR	13.8	NR
*KRAS* + *STK11* + *KEAP1* + *TP53*	33	0.8	8.6	3.96	15.12
*KEAP1*-only	25	0.6	21.1	3.96	41.64

(Co-)mutations listed in rows are sorted according to their frequency. Quadruple negative signifies that within the tumor, no mutations in the genes of *KRAS*, *STK11*, *KEAP1*, and *TP53* were found. CI, confidence interval; LUAD, lung adenocarcinoma, mOS, median overall survival; N, number of patients, NR, not reached.

Statistical analyses were performed in Python (v.3.9.). All p-values were corrected for multiple testing using false discovery rates (q-value) and q-values < 0.05 were defined as significant. The Kaplan-Meier method was performed to calculate OS curves and medians. Pairwise differences were calculated by log-rank tests.

## 3 Results

### 3.1 Demographics and incidences of (co-)mutations in NSCLC and LUAD

The total dataset consisted of 4,855 NSCLC patients (male: 41.7%; female: 58.3%). Thereby, 84.6% (N = 4,109) were lung adenocarcinoma, 11.2% (N = 542) lung squamous cell carcinoma, and 4.2% (N = 204) other histologic types of lung cancer: e.g. adenosquamous carcinoma, sarcomatoid lung cancer, lung neuroendocrine tumors (large cell neuroendocrine carcinoma, carcinoids), or not otherwise specified NSCLC. For the following analyses we concentrated on lung adenocarcinoma data. Thereby, we found the following mutation frequencies: 45.6% *TP53*, 34.17% *KRAS*, 16.82% *STK11*, 14.35% *KEAP1*. Further, *KRAS* mutations showed the subsequent distribution of point mutations: G12C 40.17%, G12V 17.32%, G12D 13.76%, G12A 7.59%, Q61H 4.26%, G13C 3.62%.

*TP53*-only mutation (29%; N = 1193), the absence of the four mutations (quadruple-negative: 25.9%; N = 1,062) and *KRAS*-only (13.4%; N = 552) were the most prevalent (co-)mutational patterns. The least prevalent (co-)mutational patterns were *KEAP1*-only (0.6%; N = 25) and the quadruple mutation (*KRAS* + *STK11* + *KEAP1* + *TP53*; 0.8%; N = 33). Full data are shown in **Table 1** and **Figure 1**.

While mutations in *KRAS*, *STK11* and *KEAP1* significantly co-occurred among themselves (q < 0.05), there was neither a significant co-occurrence nor a significant mutual exclusivity between mutations in one of the three genes and *TP53* mutations.

### 3.2 *TP53* mutations influence overall survival for better or worse depending on co-mutations

**Figure 3:**
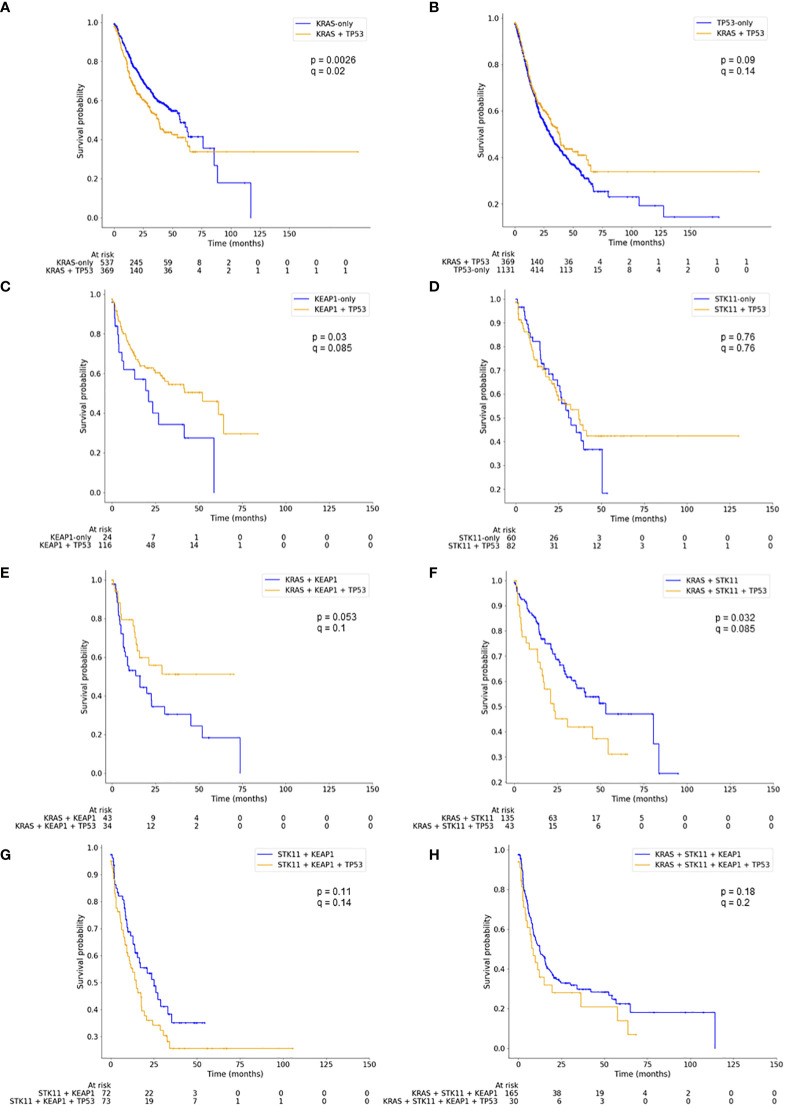
Kaplan Meier curves showing pairwise comparison of mutation groups with (blue) and without (yellow) additional *TP53* mutation. Log-rank tests were performed (p-values) and p-values corrected for multiple testing using false discovery rates (q-values). Panel A compares *KRAS*-only with *KRAS*+*TP53*, panel B *TP53*-only with *KRAS*-*TP53*, panel C *KEAP1*-only with *KEAP1*+*TP53*, panel D *STK11*-only with *STK11*+*TP53*, panel E *KRAS*+*KEAP1* with *KRAS*+*KEAP1*+*TP53*, panel F *KRAS*+*STK11* with *KRAS*+*STK11*+*TP53*, panel G *STK11*+*KEAP1* with *STK11*+*KEAP1*+*TP53*, and panel H *KRAS*+*STK11*+*KEAP1* with *KRAS*+*STK11*+*KEAP1*+*TP53*.

Kaplan-Meier curves were calculated for all 16 mutation groups and are shown in **Figure 2**. Quadruple negative (mOS = 64 months), *KRAS*-only (56.5 months) and *KRAS* + *STK11* (mOS = 53 months) mutated patients had the longest mOS, while patients mutated in *KRAS* + *STK11* + *KEAP1* + *TP53* (mOS = 8.6 months), *KRAS* + *STK11* + *KEAP1* (mOS = 12.4 months) and *STK11* + *KEAP1* + *TP53* (mOS = 14.8 months) showed the shortest mOS (**Figure 2**, **Table 1**). To determine the influence of *TP53* co-mutations on *KRAS*-, *STK11*- and/or *KEAP1*-mutated LUAD, pairwise tests were performed (**Figure 3**).

Co-mutations in *TP53* led to significantly reduced mOS in LUAD patients harboring only a *KRAS* mutation (mOS: 56.5 vs. 38.3 months; p = 0.0026; q = 0.021; **Figure 3A**) or a co-mutation in *KRAS* + *STK11* (mOS: 53.0 vs. 23.0 months; p = 0.032; q = 0.085; **Figure 3E**), albeit, significance for the latter does not remain after correcting for multiple testing. The well-known negative impact of the **KRAS* + *STK11* + *KEAP1** (mOS: 12.4 months) and *STK11* + *KEAP1* mutation co-occurrence (mOS: 25.1 months) worsened mOS by trend through an add-on *TP53* mutation (mOS: 8.6 or 14.8 months, respectively; **Figures 3G, H**); however, not statistically significant.

In contrast, concurrent *TP53* mutations to *KEAP1*-only and to *KRAS* + *KEAP1* mutations showed an opposite effect and led to an improved mOS: 21.1 months for *KEAP1*-only improved to 52.2 months for *KEAP1* + *TP53* (p = 0.03; q = 0.085; **Figure 3C**) and 16.1 months for *KRAS* + *KEAP1* improved to a mOS which did not reach the median for *KRAS* + **KEAP1* + *TP53** (p = 0.053; q = 0.1; **Figure 3F**).

When considering only *KRAS* mutations harboring the G12C alteration, the add-on *TP53* mutation still led to reduced mOS (85.7 vs. 36.5 months; p = 0.02; q = 0.08), albeit without significance after correcting for multiple testing, while for *KRAS* (G12C) *+ *KEAP1**, the *TP53* co-mutation still did not significantly change mOS (20 months vs. NR; p-value = 0.25; q = 0.5) (**Table 2**). The occurrence of *TP53* co-mutation in *KRAS* (G12C) *+ *STK11** altered LUAD did not lead to a reduced mOS anymore (49 vs. 54 months; p = 0.87; q = 0.87). This is also true for *KRAS* G12C + *STK11* + *KEAP1* (18.7 vs. 8.6 months; p = 0.48; q = 0.64). These results must be interpreted with caution due to partly small group sizes (N < 20; **Table 2**).

**Table 2:** Log-rank test comparing overall survival of *KRAS* G12C mutation groups with and without *TP53* co-mutation.

Group 1			Group 2 (with *TP53* mutation)			Statistics		
Mutations	N	mOS (months)	Mutations	N	mOS (months)	p-value	Reject	FDR p-value
*KRAS* G12C only	207	85.7	*KRAS* G12C + *TP53*	157	36.5	0.02	false	0.08
*KRAS* G12C + *KEAP1*	14	20.0	*KRAS* G12C + *KEAP1* + *TP53*	16	NR	0.25	false	0.50
*KRAS* G12C + *STK11*	71	49.0	*KRAS* G12C + *STK11* + *TP53*	17	54	0.87	false	0.87
*KRAS* G12C + *STK11* + *KEAP1*	72	18.7	*KRAS* G12C + *STK11* + *KEAP1* + *TP53*	12	8.6	0.48	false	0.64

FDR, false discovery rate; mOS, median overall survival; N, number of patients; NR, not reached.

## 4 Discussion

Here, we presented a comparative bioinformatic analysis referring to data of 4,109 patients with the aim of analyzing the influence of *TP53* mutations in *KRAS*, *STK11* and *KEAP1* (co-) mutated LUAD on the patients’ overall survival. By employing this database approach, we were able to show that *TP53* mutations had an influence on mOS for better or worse depending on the concurrent mutational pattern.

Therapy and prognosis of NSCLC has changed in the last 15 years as several treatable targets have been detected within the concept of so-called personalized therapies. In daily practice, these targets encompass testing for rearrangements (*ALK*, *ROS*, *RET*, *NTRK, MET*) and mutations (*KRAS*, *EGFR*, *BRAF*, *ERBB2*) (18–20). For a long time *KRAS*-mutations were called “untreatable targets”. Since early 2022 a specific (second-line) therapy for *KRAS* G12C has been available in Europe. However, the majority of NSCLC do not harbor the above-mentioned mutations and thus do not qualify for these treatment options. Thus, therapy is still based on different chemotherapy protocols with or without ICIs.

In our study, the mutation frequencies of the four genes (*TP53*: 45.6%, *KRAS*: 34.17%, *STK11*: 16.82%, *KEAP1*: 14.35%) correspond to the generally described values in LUAD (6). Further, as already shown (5), *KRAS*-only and *KRAS* + *STK11* mutated patients have the longest mOS (56.5 and 53 months). This seems also true for the quadruple negative group as presented here (64 months). Nevertheless, it must be noticed that the overall survival of quadruple negative patients might be biased due to further common mutations or genetic rearrangements in genes like *EGFR*, *ALK* or *ROS1* and their already approved targeted therapies. However, the comparable long survival times of these groups, especially for *KRAS*-only, further underlines the fact that the time has passed to describe *KRAS* as a prognostically unfavorable factor. Moreover, it seems more appropriate to analyze the complex surrounding mutational landscape, as patients mutated in *KRAS* + *STK11* + *KEAP1* + *TP53*, *KRAS* + *STK11* + *KEAP1* and *STK11* + *KEAP1* + *TP53* show the shortest overall survival. So far, several studies described *STK11* and *KEAP1* alone or co-mutated with *KRAS* having a negative impact on OS, response to ICI-therapy (4, 5, 21–26) and also across treatment classes independent of immunotherapy (4, 5, 24, 27). However, especially *KEAP1* mutations seemed to be the driving factor being the only one significant in a multivariate model (4). This is also reflected in the current analyses of the CodeBreak 100 clinical trial. Here, *KEAP1* mutations also appear to be a negative prognostic marker for sotorasib (28).

Therefore, it is particularly interesting that this role seems only true if *TP53*-mutations are absent, as patients with the combination of **KRAS* + *KEAP1* + *TP53** or **KEAP1* + *TP53** co-mutations show an improved mOS in comparison to *KEAP1*-only and *KRAS* + *KEAP1* mutated patients. Thus, somewhat surprisingly, in this setting *TP53* mutations seem to have a protective effect as long as *STK11* is not co-mutated. So far, survival advantage of *TP53* mutations could be shown under therapy with ICI (9, 11–13), however not across treatments. Regardless, it must be noticed that these studies did not include *KEAP1* co-mutations into their analyses.

Vice versa, *TP53* will have a negative influence on **KRAS*-only* mutated LUAD. Here, the patients’ mOS was reduced significantly more than 30% (from 56.5 to 38.3 months, see **Table 1**, **Figure 3A**). The negative influence of *TP53* mutations were also visible for the following co-mutations: *KRAS* + *STK11*, *KRAS* + *STK11* + *KEAP1* and *STK11* + *KEAP1* (**Figure 3**). Therefore, the potential positive or negative impact of the altered tumor suppressor p53 seems to depend on the surrounding mutational network. This effect had already been described by Saleh et al. and Scalera et al., pointing out that molecular stratification of both alterations should be implemented for localized and advanced-stage NSCLC to optimize and modify clinical decision-making (29, 30), even though both studies did not include *KRAS* and/or *STK11* in their investigations.

For *KRAS* G12C, sotorasib, a targeted therapy, is approved and has shown that its efficacy is influenced by the co-mutations of *STK11* and *KEAP1*. While the co-mutation with *STK11* leads to a slightly improved efficacy, *KEAP1* and the co-mutation with both genes result in a reduced response (31). Therefore, we performed our analyses in the context of *KRAS* G12C. Comparable results were observed with a reduced overall survival when *KRAS* G12C (mOS = 85.7 month) is co-mutated with *TP53* (mOS = 36.5; p = 0.02; q = 0.08). The benefits of *TP53* co-mutations shown under ICI were subsequently not apparent across treatments (8, 9). The reported tendency was not shown in the context of **KRAS* G12C + *STK11* + *TP53*.* Here again, a somewhat protective effect might be discussed for *TP53* co-mutations in *KRAS* G12C + *KEAP1* mutated patients, albeit group sizes are small and results not significant. This underlines the importance of the now available G12C-targeted therapy and the need for more druggable options in *KRAS*-mutated LUAD and should be kept in mind when interpreting the results of the recently published phase III CodeBreaK 200 trial (32) and the still ongoing phase III CodeBreaK 202 trial (NCT05920356) evaluating sotorasib for the second-line or first-line treatment, respectively.

Finally, we here present a bioinformatic analysis of merged data sets. This might be a limitation at first sight, as we did not refer to our own data. However, the sample sizes of the molecular subgroups, as summarized here, are too small within the single studies to obtain group sizes sufficient for robust statistical results. Thus, integrating database approaches, as presented here, are needed to draw first preliminary conclusions and to generate new hypotheses. These hypotheses must be then tested in future multi-center investigations. This study is further limited by the given annotations. Analyzing the mutation groups in correlation with further clinical variables like age, sex, smoking history, and in particular different treatment patterns is an important task for future studies. Another point of interest is the assessment of progression-free survival in addition to the overall survival. Nevertheless, our study gives important insights into the mutual influence of co-mutations and provides a starting point for future research approaches.

To conclude, the more mutations are analyzed to a greater extent, the greater will be the complexity of the mutational network of lung cancer and cancer in general. In the daily clinical routine setting, referring to panel-based sequencing (as e.g. suggested by the national network of genomic medicine/nNGM) seems mandatory and focusing on different combinations of mutations can help define different prognostic groups and might be the starting point for new treatment strategies.

## Data availability statement

The original contributions presented in the study are included in the article/supplementary material. Further inquiries can be directed to the corresponding authors.

## Ethics statement

Ethical approval was not required for the study involving humans in accordance with the local legislation and institutional requirements. Written informed consent to participate in this study was not required from the participants or the participants’ legal guardians/next of kin in accordance with the national legislation and the institutional requirements.

## Author contributions

AF: Conceptualization, Data curation, Formal Analysis, Funding acquisition, Investigation, Methodology, Project administration, Resources, Software, Supervision, Validation, Visualization, Writing – original draft, Writing – review & editing. MB: Conceptualization, Data curation, Formal Analysis, Funding acquisition, Investigation, Methodology, Project administration, Resources, Software, Supervision, Validation, Visualization, Writing – original draft, Writing – review & editing. HW: Writing – review & editing. MS: Methodology, Writing – review & editing. HB: Funding acquisition, Supervision, Writing – review & editing. ML: Conceptualization, Data curation, Formal Analysis, Funding acquisition, Investigation, Methodology, Project administration, Resources, Software, Supervision, Validation, Visualization, Writing – original draft, Writing – review & editing.
