# Corrigendum: *TP53* co-mutations in advanced lung adenocarcinoma: comparative bioinformatic analyses suggest ambivalent character on overall survival alongside *KRAS*, *STK11* and *KEAP1* mutations

**DOI:** 10.3389/fonc.2024.1473239

**Published:** 2024-09-11

**Authors:** Armin Frille, Myriam Boeschen, Hubert Wirtz, Mathias Stiller, Hendrik Bläker, Maximilian von Laffert

**Affiliations:** ^1^ Department of Respiratory Medicine, Leipzig University, Leipzig, Germany; ^2^ Institute of Pathology, Leipzig University, Leipzig, Germany

**Keywords:** NSCLC, lung adenocarcinoma, *KRAS*, *STK11*, *KEAP1*, *TP53*, co-mutations, survival

In the published article, there was a mistake in the **Funding** statement. The original text of the **Funding** statement was unspecific, since financial support was received for the publication costs only.

The first two sentences of the original **Funding** statement read:

“The author(s) declare financial support was received for the research, authorship, and/or publication of this article. This publication was funded by the Open Access Publishing Fund of Leipzig University, supported by the German Research Foundation (DFG).”

The correct **Funding** statement appears below.

**Funding**

“The authors declare that financial support was received for the publication of this article, which was funded by the Open Access Publishing Fund of Leipzig University, supported by the German Research Foundation (DFG). The funder was not involved in the study design, collection, analysis, interpretation of data, the writing of this article or the decision to submit it for publication.”

In the published article, there was an error in the **Abstract** section.

The words “in patients with lung cancer” were added after the term “(mOS)”.

**Abstract,** *Background*. This sentence previously stated:

“Recently, we could show that the co-mutations of *KRAS* + *KEAP1*, *STK11* + *KEAP1* and *KRAS* + *STK11* + *KEAP1* lead to a significantly shorter median overall survival (mOS) across treatments by analyzing multiple datasets.”

The corrected sentence appears below:

“Recently, we could show that the co-mutations of *KRAS* + *KEAP1*, *STK11* + *KEAP1* and *KRAS* + *STK11* + *KEAP1* lead to a significantly shorter median overall survival (mOS) in patients with lung cancer across treatments by analyzing multiple datasets.”

In the published article, there was an error in the **Abstract** section.

The word “*STK1*” was replaced with “*STK11*” at the end of the sentence.

**Abstract,** *Results*. This sentence previously stated:

“Whereas TP53-mutations seem to have protective effects in the context of further *KEAP1-* and *KRAS* + *KEAP1-*alterations (improved mOS), their role seems contrary if acquired in an already existing combination of mutations as *KRAS* + *STK11*, *KRAS* + *STK11* + *KEAP1* and *STK1* + *KEAP1*.”

The corrected sentence appears below:

“Whereas TP53-mutations seem to have protective effects in the context of further *KEAP1-* and *KRAS* + *KEAP1-*alterations (improved mOS), their role seems contrary if acquired in an already existing combination of mutations as *KRAS* + *STK11*, *KRAS* + *STK11* + *KEAP1* and *STK11* + *KEAP1*.”

In the published article, there was an error in the **Abstract** section. The word “co-mutationshad” was replaced with “co-mutations had” in the last sentence of the results section.

**Abstract,** *Results*. This sentence previously stated:

“TP53 co-mutationshad a negative influence on KRAS- only mutated LUAD (mOS reduced significantly by more than 30%).”

The corrected sentence appears below:

“TP53 co-mutations had a negative influence on KRAS- only mutated LUAD (mOS reduced significantly by more than 30%).”

In the published article, there was an error in the **Introduction** section.

The word “KRAS” was italicized.

**1 Introduction,** Paragraph 5. This sentence previously stated:

“Whereas, *TP53* mutations alone had no impact on response and survival, a subgroup (12 patients) with KRAS *G12C* + *TP53* co-mutations defined long-term responders to immunotherapy (9).”

The corrected sentence appears below:

“Whereas, *TP53* mutations alone had no impact on response and survival, a subgroup (12 patients) with *KRAS G12C* + *TP53* co-mutations defined long-term responders to immunotherapy (9).”

In the published article, there was an error in the **Material and methods** section.

The word “compromise” was replaced with “comprised”.

**2 Materials and methods**, Paragraph 1. This sentence previously stated:

“All data compromise patients with advanced tumor stages (mainly stage IV).”

The corrected sentence appears below:

“All data comprised patients with advanced tumor stages (mainly stage IV).”

In the published article, there was an error in the **Results** section.

The symbol “>“ was replaced with “<“.

**3 Results**, *3.1 Demographics and incidences of (co-)mutations in NSCLC and LUAD*, Paragraph 3. This sentence previously stated:

“While mutations in *KRAS*, *STK11* and *KEAP1* significantly co- occurred among themselves (q > 0.05), there was neither a significant co-occurrence nor a significant mutual exclusivity between mutations in one of the three genes and *TP53* mutations.”

The corrected sentence appears below:

“While mutations in *KRAS*, *STK11* and *KEAP1* significantly co- occurred among themselves (q < 0.05), there was neither a significant co-occurrence nor a significant mutual exclusivity between mutations in one of the three genes and *TP53* mutations.”

In the published article, there was an error in the **Discussion** section.

The word “mutation” was replaced with “mutational pattern”.

**4 Discussion**, Paragraph 1. This sentence previously stated:

“By employing this database approach, we were able to show that *TP53* mutations had an influence on mOS for better or worse depending on the concurrent mutation.”

The corrected sentence appears below:

“By employing this database approach, we were able to show that *TP53* mutations had an influence on mOS for better or worse depending on the concurrent mutational pattern.”

The authors apologize for these errors and state that this does not change the scientific conclusions of the article in any way. The original article has been updated.

